# Optimal cultivation of *Chlamydia* requires testing of serum on individual species

**DOI:** 10.1186/s13104-020-4893-9

**Published:** 2020-01-13

**Authors:** Malhar Desai, Huirong Zhang, Huizhou Fan

**Affiliations:** 1 Department of Pharmacology, Robert Wood Johnson Medical School, Rutgers University, Piscataway, NJ USA; 2 Graduate Program in Physiology and Integrative Biology, School of Graduate Studies, Piscataway, NJ USA

**Keywords:** *Chlamydia trachomatis*, *Chlamydia muridarum*, Fetal bovine serum, Serum test

## Abstract

**Objective:**

This report is a side product of experiments aimed at identifying serum for culturing obligate intracellular bacteria *Chlamydia trachomatis* and *C. muridarum* in mouse fibroblast L929 cells.

**Results:**

Of five commercial serum samples tested, two showed optimal efficiencies at supporting growth of the human pathogen *Chlamydia trachomatis* as control fetal bovine serum, whereas two showed modest ~ 40% inhibitions in progeny production, and the remaining one showed a 20% inhibition. Three of the six sera poorly supported growth of the murine pathogen *Chlamydia muridarum*, resulting in 73–90% reduction in progeny formation. Most significantly, the one with the strongest (90%) *C. muridarum* inhibition activity showed optimal *C. trachomatis*-supporting efficiency. These findings indicate that in laboratories that study multiple *Chlamydia* species, serum samples should be prescreened on a species basis. Considering *Chlamydial* biology and epidemiology, it may even be necessary to perform serum tests on a serovar- or strain-basis for studying some animal chlamydiae.

## Introduction

Animal serum serves as a rich source of growth factors for culturing animal cells [1] and intracellular pathogens requiring animal cells as hosts (e.g., [2–5]). Fetal bovine serum (FBS), which contains a large and diverse group of growth factors, has been a popular choice. However, the high costs of FBS (due to its limited supplies) and ethical concerns associated with its production have led some researchers to pursue using other alternatives to FBS, including newborn calf serum (NBCS, defined as serum collected from calves that are less than 10 days old) and horse serum [1–5].

*Chlamydia* is a genus of bacteria that replicates only inside host eukaryotic cells [6]. *Chlamydia trachomatis* and *C. pneumoniae* are common human pathogens. Whereas *C. trachomatis* is the number one sexually transmitted pathogenic bacterium [7], *C. pneumoniae* causes communicable respiratory infections [8]. Other *Chlamydia* species known as pathogens of animals including livestock may cause severe illness in humans after their contact with infected animals [9]. *C. psittaci,* cause of psittacosis, is the best example of zoonotic *Chlamydia*. *C. muridarum* is a mouse pathogen. Although *C. muridarum* infection in humans has never been documented, it is the most widely studied animal *Chlamydia,* owing to its capacity to model human chlamydial infections in mouse [10, 11].

Like many laboratories, our laboratory studies both *C. trachomatis* and *C. muridarum.* In an event evaluating commercial serum samples, we found differential effects of the sera on the two *Chlamydia* species. These findings mandate prescreening sera for individual species in laboratories that study multiple species.

## Main text

### Methods

#### Animal serum

Samples of FBS, NBCS and ASC were supplied by American Type Culture Collection (ATCC), Atlantic Biologicals, Gemini Bio-products and Sigma Millipore. Prior to use, all serum samples as well as control FBS (Sigma Millipore) were subjected to heat inactivation (56 °C, 30 min) to eliminate adverse effects of complements on chlamydiae [12].

#### Evaluation of effects of serum on chlamydial growth

Mouse fibroblast L929 cells were used as host for chlamydiae. They were grown as adherent cultures using the Dulbecco modified Eagle’s medium containing 4.5 g/L glucose and 0.11 g/L sodium pyruvate (DMEM) and supplemented with 5% (vol/vol) control FBS and gentamicin (final concentration: 10 µg/ml). To evaluate the effects of serum samples on chlamydial growth, cells were seeded onto 6-well plates and cultured overnight to 90% confluency. Elementary body (EB) stocks of *C. trachomatis* L2 (strain 434/Bu) (CtL2) and *C. muridarum* (strain Nigg) (Cm) [13] were diluted in DMEM containing 5% serum samples and 1 µg/ml cycloheximide, which promotes chlamydial growth by inhibiting host cellular protein synthesis. To infect the cells, the overnight culture media were replaced with the EB-containing medium (2 ml/well). The multiplicity of infection was 0.5 inclusion-forming unit (IFU) per cell. After culture at 37 °C for 30 h (Cm) or 36 h (CtL2), the culture media were replaced with 100 μL of sucrose–phosphate–glutamate buffer, and cells were detached from the plastic surface using a Cell Lifter, and collected into a 5 ml culture tube. Tubes were then placed on ice and subject to brief sonication to release chlamydiae from cells [13]. The sonicated harvests were serially diluted in a 1:10 manner and then inoculated onto L929 monolayers grown on 96 well plates, which were fixed with cold methanol following 30 h (Cm) or 36 h (CtL2) incubation at 37 °C. Cm and CtL2 inclusions were reacted with a mouse polyclonal anti-Cm antibody [14] and a monoclonal anti-major outer membrane protein antibody (clone L2-1-5) [15], respectively, followed by fluorescein isothiocyanate-conjugated rabbit anti-mouse IgG (Sigma-Aldrich). Inclusions were scored under an Olympic IX51 fluorescence microscope.

## Results

**Fig. 1:**
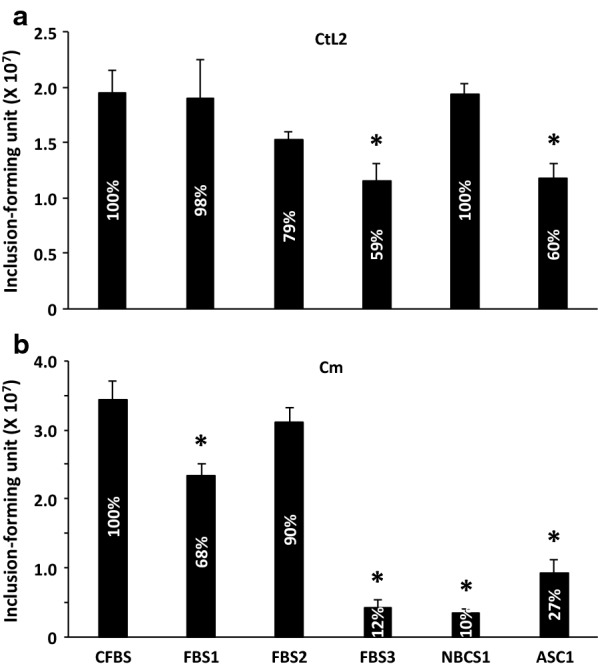
Differential seral effects on CtL2 (**a**) and Cm (**b**) growth. Averages ± standard deviations of recoverable inclusion-forming units of progeny EBs from quadruplicate tests are presented. Asterisks signify statistically significant EB yield decreases as compared to the control CFBS (*P *< 0.005)

We tested a total of 5 serum samples from commercial sources along with a control fetal bovine serum (CFBS) on the growth of *C. trachomatis* L2 (CtL2) and *C. muridarum* (Cm). Among the five serum samples, three were fetal bovine sera (i.e., FBS1-3), one was a newborn calf serum (i.e., NBCS1), and the last one was known as animal serum complex (i.e., ASC1), of which the compositions were not disclosed by the manufacturer. CFBS had been tested previously and used for culturing both Ct and Cm for more than a year in our lab prior to this work. Compared with CFBS, FBS1 or NBCS1 demonstrated similar and the most desirable effect on CtL2 growth, resulting in the highest EB yields (Fig. 1a). FBS2 showed a slight 21% decrease in EB production, which was deemed statistically insignificant (*P *= 0.009). FBS3 and ASC1 demonstrated suboptimal effects on chlamydial growth, resulting in statistically significant 41% and 40% decreases, respectively, in EB production (Fig. 1a).

Compared to CtL2, Cm displayed mostly contrasting responses to the serum products (Fig. 1b). Among all samples tested, only FBS2 showed a similar effect on Cm growth as CFBS. FBS1, which was optimal for CtL2 growth, was suboptimal for Cm, resulting in only 68% progeny EBs, compared to CFBS. Most interestingly, NBCS1, which was optimal for CtL2 growth, had the most detrimental effect on Cm growth, resulting in a 90% EB production inhibition. FBS3, which had a suboptimal effect on CtL2 growth, displayed a similarly severe adverse effect as NBCS1, leading to an 88% reduction in EB production. ASC1, which had a suboptimal effect on CtL2 growth (40% inhibition in EB yield), exhibited a more severe adverse effect on Cm growth, giving rise to a 73% decrease.

## Discussion

The importance of serum in culturing chlamydiae was recognized starting more than four decades ago (e.g., [2, 3]). However, previous studies reporting effects of different serum products on chlamydial growth were performed with only single species [2–5]. In this work, we have demonstrated that some animal sera have significantly different effects on Ct and Cm growth. Although we have not determined the underlying mechanism, we suspect anti-chlamydia antibodies are responsible for the observed inhibitory effects. Ct and Cm are known to infect only human and mouse, respectively. However, multiple other *Chlamydia* species, namely *C. pecorum* and *C. abortus, C. pneumoniae, C. psittaci* and *C. gallinacean* are known to infect cows [9, 16]. Thus, it should not be surprising if FBS or NBCS contains anti-chlamydia antibodies because maternal IgG is capable of entering the fetal blood stream. Although genomes of *Chlamydia* species are highly conserved, they express different polymorphic proteins on the cell surface. These proteins serve as immunodominant antigens, which induce hosts to produce neutralizing antibodies [17, 18]. Other outer membrane proteins including the major outer membrane protein are also immunodominant proteins [19]. Thus, the effects of a particular serum on Ct and Cm would be determined by antibodies with cross reactivity for the organisms (or not).

Surface-exposed domains of the major outer membrane protein are immunodominant epitopes [20, 21]. They are highly variable even within the same *Chlamydia* species [20, 21]. While our findings mandate serum testing for individual *Chlamydia* species for optimal cultivation, tests may be necessary for individual serovars of *Chlamydia* species capable of infecting cows. Perhaps, serum testing should be performed for individual strains of *Chlamydia* species without well-established serovars.

## Limitations

We make the recommendation of serum testing on individual *Chlamydia* species on the basis of data presented in Fig. 1. However, the suggestion of serum testing for different serovars or strains within some animal chlamydial species is made without experimental evidence.

## Data Availability

The dataset used for this study is available from the corresponding author on request.

## Abbreviations

ASC: animal serum complex
CF: control fetal bovine serum
Cm: *Chlamydia muridarum*
CtL2: *Chlamydia trachomatis* serovar L2
DMEM: Dulbecco’s Modified Eagle Medium
FBS: fetal bovine serum
IFU: inclusion forming unit
NBCS: newborn calf serum
